# Recombinant LSDV Strains in Asia: Vaccine Spillover or Natural Emergence?

**DOI:** 10.3390/v14071429

**Published:** 2022-06-29

**Authors:** Frank Vandenbussche, Elisabeth Mathijs, Wannes Philips, Meruyert Saduakassova, Ilse De Leeuw, Akhmetzhan Sultanov, Andy Haegeman, Kris De Clercq

**Affiliations:** 1 EURL for Diseases Caused by Capripoxviruses, Scientific Directorate Infectious Diseases in Animals, Sciensano, Groeselenberg 99, B-1180 Brussels, Belgium; frank.vandenbussche@sciensano.be (F.V.); elisabeth.mathijs@sciensano.be (E.M.); wannes.philips@sciensano.be (W.P.); 2 Kazakh Scientific Research Veterinary Institute (KazSRVI/KazNIVI), Raiymbek ave. 223, Almaty 050016, Kazakhstan; mika.kaznivi@gmail.com (M.S.); akhmetzhan.sultanov@gmail.com (A.S.); 3 Unit of Exotic and Particular Diseases, Scientific Directorate Infectious Diseases in Animals, Sciensano, Groeselenberg 99, B-1180 Brussels, Belgium; ilse.deleeuw@sciensano.be (I.D.L.); andy.haegeman@sciensano.be (A.H.)

**Keywords:** lumpy skin disease virus, homologous vaccine, live attenuated vaccine, spillover, recombination

## Abstract

From 2017 to 2019, several vaccine-like recombinant strains of lumpy skin disease virus (LSDV) were discovered in Kazakhstan and neighbouring regions of Russia and China. Shortly before their emergence, the authorities in Kazakhstan launched a mass vaccination campaign with the Neethling-based Lumpivax vaccine. Since none of the other countries in the affected region had used a homologous LSDV vaccine, it was soon suspected that the Lumpivax vaccine was the cause of these unusual LSDV strains. In this study, we performed a genome-wide molecular analysis to investigate the composition of two Lumpivax vaccine batches and to establish a possible link between the vaccine and the recent outbreaks. Although labelled as a pure Neethling-based LSDV vaccine, the Lumpivax vaccine appears to be a complex mixture of multiple CaPVs. Using an iterative enrichment/assembly strategy, we obtained the complete genomes of a Neethling-like LSDV vaccine strain, a KSGP-like LSDV vaccine strain and a Sudan-like GTPV strain. The same analysis also revealed the presence of several recombinant LSDV strains that were (almost) identical to the recently described vaccine-like LSDV strains. Based on their InDel/SNP signatures, the vaccine-like recombinant strains can be divided into four groups. Each group has a distinct breakpoint pattern resulting from multiple recombination events, with the number of genetic exchanges ranging from 126 to 146. The enormous divergence of the recombinant strains suggests that they arose during seed production. The recent emergence of vaccine-like LSDV strains in large parts of Asia is, therefore, most likely the result of a spillover from animals vaccinated with the Lumpivax vaccine.

## 1. Introduction

Lumpy skin disease (LSD) is an important viral disease of cattle and water buffalo. The disease is caused by the lumpy skin disease virus (LSDV), which belongs to the *Capripoxvirus* (CaPV) genus within the *Poxviridae* family [1]. The LSDV genome consists of a linear double-stranded DNA molecule approximately 150 kb in length and has a nucleotide homology of over 96% with the genomes of other members of the CaPV genus: goatpox virus (GTPV) and sheeppox virus (SPPV) [2,3]. Since the different CaPV species cannot be distinguished either morphologically or serologically, reliable differentiation is only possible at the molecular level. Several PCR assays have been developed for the detection and differentiation of CaPVs, all targeting specific nucleotide polymorphisms [4,5]. Although these PCR assays allow the identification of the different CaPV species, they can only provide a very limited snapshot of the total genetic variation and lack the discriminatory power to distinguish closely related or recombinant strains [6]. Differentiation at the strain level requires a much higher resolution that can only be achieved by comparing (nearly) complete genome sequence data using high-throughput sequencing (HTS) [7].

Lumpy skin disease was originally confined to sub-Saharan Africa but gradually spread across most of the continent. In the last decade, the disease has moved eastwards and is currently endemic in parts of the Middle East and Turkey. In August 2015, LSD emerged in Greece near the Turkish border, from where it spread across the Balkan region in 2016. Around the same time, several outbreaks were reported in the Caucasus region and Kazakhstan. Since 2019, LSD has spread eastwards and is currently present in large parts of Asia.

Because of its great economic importance, LSD is included in the list of notifiable diseases by the World Organisation for Animal Health (OIE) [8]. As with most viral diseases, there are currently no effective antiviral treatments. Control and eradication of the disease rely mainly on a combination of movement restrictions and mass vaccination [9]. Although inactivated vaccines have been described, almost all commercially available vaccines are based on live attenuated CaPV strains that induce protective immunity against LSDV [10].

Since CaPVs are antigenically indistinguishable, both homologous (i.e., LSDV-based) and heterologous (i.e., GTPV- or SPPV-based) vaccines are used worldwide to control LSD. Most homologous vaccines contain the Neethling strain, which has been attenuated by a large number of passages in cell cultures and the chorioallantoic membrane of embryonated chicken eggs [11]. Neethling-based vaccines generally provide good protection against virulent LSDV strains but can cause mild adverse reactions [10,12,13,14,15,16]. Other homologous vaccines contain the so-called Kenyan sheep and goat pox (KSGP) strains, which were later found to be LSDV strains [17,18]. Although KSGP-based vaccines have been successfully used against SPPV and GTPV, several studies have shown that these vaccines can cause clinical signs in vaccinated cattle [10,12,15]. The more pronounced adverse reactions in cattle are probably due to the relatively small number of passages used for attenuation. For economic and/or safety reasons, several countries favour the use of heterologous vaccines to control the spread of LSDV. Recent studies indicate that GTPV- and LSDV-based vaccines offer the same protection against LSDV [19,20]. Despite their high genetic similarity, SPPV-based vaccines appear to provide only partial cross-protection [21,22,23,24,25]. In contrast to LSDV-based vaccines, SPPV- and GTPV-based vaccines usually do not cause side effects in cattle.

Although Neethling-based vaccines have been used successfully for many decades, their safety has recently been questioned after several vaccine-like recombinant strains were discovered in Russia [26,27]. Whole-genome sequencing (WGS) analyses have shown that the LSDV strains from the 2015–2016 epidemics in south-eastern Europe, Kazakhstan and Russia are most similar to a strain isolated in Israel in 2012 [28,29,30,31]. In contrast, most of the recent LSDV strains from Russia and Asia appear to be vaccine-like recombinant strains carrying genetic signatures from both Neethling- and KSGP-based LSDV vaccines [26,32,33,34,35]. It is noteworthy that while the use of homologous LSDV vaccines is not authorised in Russia, the Lumpivax vaccine (KEVEVAPI) was used in Kazakhstan shortly before the emergence of the vaccine-like strains [36]. Although the route of introduction into Russia remains unclear, Sprygin et al. suggested that it was most likely due to the illegal use of the Lumpivax vaccine in Russia or the illegal movement of vaccinated animals from Kazakhstan [37]. These findings are in sharp contrast to what has been observed in the Balkan region [13,38]. Despite the annual vaccination of 1.8 million cattle with Neethling-based LSDV vaccines [39], no outbreaks caused by vaccine strains have been reported in the region or in any of the neighbouring countries [10]. Unfortunately, unlike other commercially available Neethling-based vaccines, not much is known about the Lumpivax vaccine. Although the vaccine is certified by the manufacturers KEVEVAPI (Nairobi, Kenya) and AU-PANVAC (Debre Zeit, Ethiopia) as containing the LSDV Neethling strain, a preliminary genetic characterisation by Haegeman et al. [40] revealed the presence of both Neethling- and KSGP-like LSDV sequences. In addition, the authors also discovered typical GTPV sequences [40]. Even though these results raise serious concerns about the exact contents of the vaccine, it remains unclear whether the vaccine-like recombinant strains were already present in the vaccine or only emerged in the field. In the present study, we therefore performed a more detailed molecular characterisation of the Lumpivax vaccine. The aim of the study was twofold: (1) to analyse the composition of two batches of the Lumpivax vaccine and (2) to investigate a possible link between the vaccine and the recent vaccine-like recombinant LSDV strains. Our results suggest that the Lumpivax vaccine, although labelled as a Neethling-based vaccine, contains at least three CaPV strains: a Neethling-like strain, a KSGP-like strain and a GTPV strain. In addition, the proportion of the three viruses varies depending on the batch analysed. It was possible to recover all recent vaccine-like recombinant LSDV genomes from the sequencing data, indicating that the exchange of genetic material did not occur in co-infected animals but during vaccine production.

## 2. Materials and Methods

### 2.1. Comparison of Published Recombinant LSDV Genomes

All available vaccine-like recombinant LSDV genomes were retrieved from GenBank on 31 January 2022. The recombinant strains were originally isolated in Russia (MH646674.1: LSDV/Russia/Saratov/2017, MT134042.1: LSDV/Russia/Udmurtiya/2019), Kazakhstan (MT992618.1: KZ-Kostanay-2018), China (MW355944.1: China/GD01/2020), Taiwan (OL752713.1: LSDV/KM/Taiwan/2020), Vietnam (MZ577073.1: 20L42_Quyet-Thang/VNM/20, MZ577074.1: 20L43_Ly-Quoc/VNM/20, MZ577075.1: 20L70_Dinh-To/VNM/20, MZ577076.1: 20L81_Bang-Thanh/VNM/20) and Hong Kong (MW732649.1: LSDV/HongKong/2020). The putative parental genomes, the Neethling-Herbivac vaccine (KX764644.1) and KSGP-O240 (KX683219.1), were included in the dataset to depict the characterisation of the different recombination patterns. The resulting dataset (*n* = 12) was aligned with MAFFT v.7.490 using the FFT-NS-i algorithm [41]. The patterns of DNA exchange in the recombinant genomes were visualised using the visual summary report of Base-By-Base [42]. The output of the multiple genome comparison statistics from Base-By-Base was used to study the breakpoints in more detail. Single-nucleotide polymorphisms (SNPs) detected in the recombinant genomes were ascribed as originating from either one of the parental genomes. Regions where the SNP pattern switches from one parental genome to another are identified as potential recombination breakpoints.

### 2.2. Vaccine Batches

Two batches of Lumpivax^TM^ with 100 doses each (KEVEVAPI, Nairobi, Kenya) were used: batch No. 05/2017 (B-0517) and batch No. 02/2019 (B-0219). Both vials were labelled as “A freeze-dried live attenuated vaccine prepared from the Neethling strain” (https://kevevapi.or.ke/lumpivax/, accessed on 4 March 2022).

### 2.3. DNA Purification

The freeze-dried vaccine pellets were dissolved in 2 mL phosphate-buffered saline. Two hundred and forty microliters of the sample was treated with 30 MBU of Baseline Zero DNase (Lucigen, Middleton, WI, USA) prior to DNA extraction. High-molecular-weight DNA was obtained with a Puregene Core Kit A (Qiagen, Hilden, Germany) according to the manufacturer’s instructions. Briefly, the sample was lysed and treated with proteinase K and RNase. After the removal of contaminating proteins, DNA was precipitated, and the pellet was resuspended in 100 µL of 10 mM Tris-HCl, pH 8.5.

### 2.4. Pre-Sequencing Enrichment

In order to achieve uniform coverage of the entire genome, an enrichment step was performed prior to sequencing using an in-house long-range PCR that covered the entire genome with 23 overlapping amplicons of approximately 7.5 kb in length [43]. Briefly, PCRs were performed in a mix containing 1 M betaine, 0.5 μM of both forward and reverse primers, 0.4 mM CleanAmp dNTPs (TriLink Biotechnologies, San Diego, CA, USA) and 1 U of Q5 High-Fidelity DNA polymerase (New England Biolabs, Ipswich, MA, USA). Cycling conditions were as follows: 98 °C for 3 min, 35 cycles of 10 s at 98 °C, 30 s at 63 °C and 7 min at 72 °C, followed by 2 min at 72 °C. Each amplicon was individually visualised by gel electrophoresis and purified using the Agencourt AMPure XP system (Beckman Coulter, Brea, CA, USA). The purified DNA was quantified and equimolarly pooled.

### 2.5. Library Preparation and Sequencing

DNA shearing, KAPA HyperPrep library preparation and MiSeq sequencing were performed at the Neuromics Support Facility (VIB-UAntwerp Centre for Molecular Neurology, Antwerp, Belgium). Briefly, the equimolar amplicon pools were fragmented by sonication to an average size of 500 bp using a Bioruptor (Diagenode, Seraing, Belgium). For B-0517 and B-0219, libraries were prepared using a KAPA HyperPrep Kit (Roche, Basel, Switzerland) according to the manufacturer’s instructions. The resulting data are further referred to as B-0517_PCR and B-0219_PCR. An additional library (B-0517_DNA) was prepared directly from 0.8 ng of purified B-0517 DNA (i.e., without PCR enrichment) using a Nextera XT DNA Library Preparation Kit (Illumina, San Diego, CA, USA). All libraries were sequenced on a MiSeq System using the MiSeq Reagent Kit version 3, 2 × 300 bp (Illumina, San Diego, CA, USA).

### 2.6. Sequencing Data Processing

The quality of the raw data was assessed using FastQC v.0.11.7 [44]. Adapter sequences and low-quality bases were removed with Trimmomatic v.0.38 using the MAXINFO adaptive quality trimming criterion [45]. Primer sequences were removed from the B-0517_PCR and B-0219_PCR datasets using BBDuk v.38.93 [46]. The trimmed reads were mapped against CaPV genomes using BBMap v.38.93 [46] run either in normal mode (NC_003027: Neethling NI-2490) or in perfect mode (KX764644: LSDV-Herbivac, KX683219: LSDV-KSGP-O240, MN072624: GTPV-Sudan). Coverage depth across the genomes was plotted in R using the ggplot2 package (v.4.0.2) [47].

### 2.7. De Novo Assembly without In Silico Enrichment

The trimmed reads were de novo assembled using SPAdes v.3.15.2 [48] with optimised k values and a subsample of 40,000 paired-end reads as described previously [43]. The quality of the de novo assemblies was assessed and compared using Quast v.5.1.0 [49].

### 2.8. De Novo Assembly with In Silico Enrichment

The genomes of the different CaPV strains were reconstructed using an iterative strategy involving several rounds of in silico enrichment and de novo assembly. Reads were mapped against a reference dataset using BBMap v.38.93 [46] in perfect mode (first round only) and semi-perfect mode (all later rounds). The mapped reads were subsequently assembled into CaPV contigs using SPAdes v.3.15.2 [48] in isolate mode, and the resulting contigs were manually stitched together into one or more scaffolds by replacing the missing positions with N’s. The scaffolds were added to the previous reference dataset, and the entire process was repeated until a complete CaPV genome was obtained.

The GTPV genome was reconstructed from the B-0219_PCR dataset using a GTPV subgroup 2.3 dataset comprising the genomes of GTPV-Oman (MN072623), GTPV-Sudan (MN072624) and GTPV-Yemen (MN072625) [50]. The LSDV genomes from the putative parental strains and the vaccine-like recombinant strains were assembled from the B-0517_PCR dataset using the respective LSDV genomes as a reference dataset.

## 3. Results

### 3.1. Data Output of the Different Sequencing Libraries

The data output of the different sequencing libraries is summarised in Table 1. MiSeq sequencing yielded a similar number of reads for all libraries, with over 3,000,000 paired-end reads remaining after quality trimming. More than 99% of the reads obtained after PCR enrichment (B-0517_PCR and B-0219_PCR) mapped to the LSDV reference genome, which was almost completely covered. Although no enrichment was performed, no less than 83.29% of the B-0517_DNA reads were of LSDV origin, resulting in complete coverage of the reference genome. Most of the remaining reads were of bacterial origin (data not shown).

### 3.2. Composition of the Lumpivax Vaccine

In a first attempt to characterise the Lumpivax vaccine strain, we performed a standard de novo assembly using a subset of the trimmed reads from B-0517_PCR and B-0219_PCR. Despite the large number of reads mapping to the LSDV reference genome and in contrast to previous studies [18,34,51], we were unable to reconstruct a complete genome from either batch. Instead, we obtained a large number of relatively short contigs. To better understand these unexpected results, the assemblies of both batches were compared with QUAST using a typical Neethling-based vaccine strain (i.e., LSDV-Herbivac) as a reference genome (Table 2). Although both assemblies cover over 90% of the LSDV-Herbivac genome, it was clear that neither is very accurate. Both the number of mismatches and the number of insertions/deletions (InDels) per 100 kbp were much higher than would be expected for a Neethling-based vaccine strain, as previously sequenced strains differ by only a handful of SNPs and/or InDels [51]. Interestingly, the B-0219_PCR assembly appeared to be even less accurate than the B-0517_PCR assembly, even though no clear differences in read quality were observed (Table 1). Both assemblies also exhibited strikingly different contiguity metrics, with the B-0219_PCR assembly yielding fewer but larger contigs.

**Figure 1:**
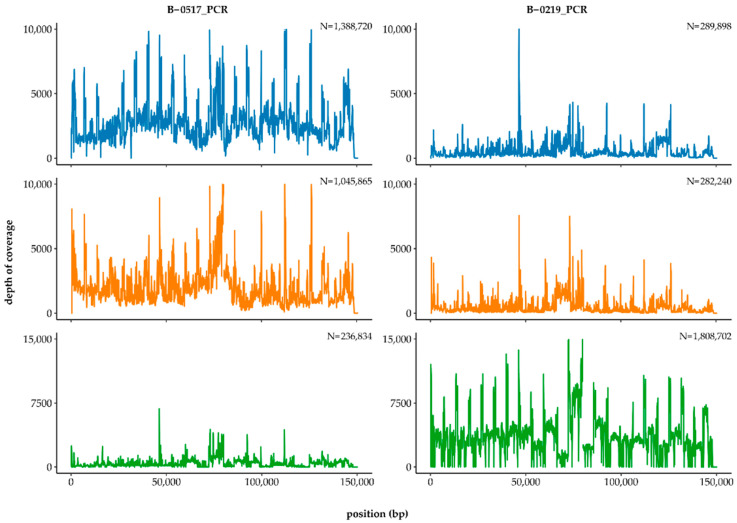
Differences in the composition of Lumpivax batches B-0517 and B-0219. Trimmed reads of both datasets were mapped to the genomes of LSDV-Herbivac (blue), LSDV-KSGP-O240 (orange) and GTPV-Sudan (green) using BBMap in perfect mode. The number of paired-end reads is indicated in the top right corner of each subplot.

The results of the assembly suggest that the Lumpivax vaccine strain is very different from other attenuated Neethling-vaccine strains and that batches B-0517 and B-0219 had different compositions. To investigate these results further, we compared the contigs of both assemblies with the GenBank database. BLAST analysis revealed that the B-0517_PCR assembly contained mainly LSDV-derived sequences belonging to either subgroup 1.1 or 1.2, whereas the majority of the B-0219_PCR contigs were GTPV-derived sequences from subgroup 2.3, according to the classification of Biswas et al. (Table S1) [50]. The unusually high divergence was thus due to the presence of multiple CaPV strains rather than a single, more distantly related LSDV strain. To investigate the composition of the two batches in more detail, reads from both datasets were mapped to the genomes of LSDV-Herbivac (CaPV subgroup 1.1), KSGP-O240 (CaPV subgroup 1.2) and GTPV-Sudan (CaPV subgroup 2.3) using BBMap in perfect mode (Figure 1). Both B-0517_PCR and B-0219_PCR contained reads that mapped perfectly to one of the three genomes, but their proportions differed markedly. Whereas B-0517_PCR mainly contained LSDV-derived reads, most of the B-0219_PCR reads were of GTPV origin.

### 3.3. Reconstruction of the CaPV Strains Present in the Lumpivax Vaccine

Since the Lumpivax vaccine appeared to be a mixture of several CaPVs, we decided to unravel its composition step by step, starting with the more divergent GTPV strain and then proceeding to the LSDV strains.

#### 3.3.1. Reconstruction of the Lumpivax GTPV Strain

To reconstruct the GTPV strain, we initially focused on the B-0219_PCR dataset, which appears to contain the most GTPV reads. Using an iterative strategy involving in silico enrichment followed by de novo assembly, we were able to obtain a nearly complete GTPV genome in two rounds (File S1). Comparison with previously published strains showed that this GTPV-Lumpivax strain was closely related to GTPV-Sudan. Nevertheless, a pairwise comparison between the two strains revealed 77 SNPs and 36 InDels. To investigate whether the new GTPV strain was present in both batches, we subsequently mapped all reads from the B-0517_PCR dataset to the GTPV-Lumpivax genome using BBSplit. Although the number of GTPV reads in this dataset was much lower, we were able to cover almost the entire genome (data not shown). These results suggest that the GTPV strain was already present in the first batch but has increased over time.

#### 3.3.2. Reconstruction of the Lumpivax LSDV Vaccine Strains

After removing the GTPV reads, we used the same strategy to recover the genomes of the Neethling- and KSGP-based vaccine strains. Based on the number of LSDV reads, we decided to use the B-0517_PCR dataset for the LSDV assemblies. The genomes of both vaccine strains were reconstructed in a single round. Since only perfectly matched reads were used in the first round, these reconstructed genomes were identical to the previously published sequences. The presence of at least two closely related strains in the same vaccine increases the possibility that the viruses exchanged genetic material during replication. The datasets were therefore further explored to confirm the presence of chimeric genomes in the Lumpivax vaccine and to investigate a possible link with the recombinant LSDV strains isolated from recent outbreaks.

### 3.4. Presence of the Vaccine-like Recombinant LSDV Strains

#### 3.4.1. Recombination Patterns of the Recombinant Field LSDV Strains

**Figure 2:**
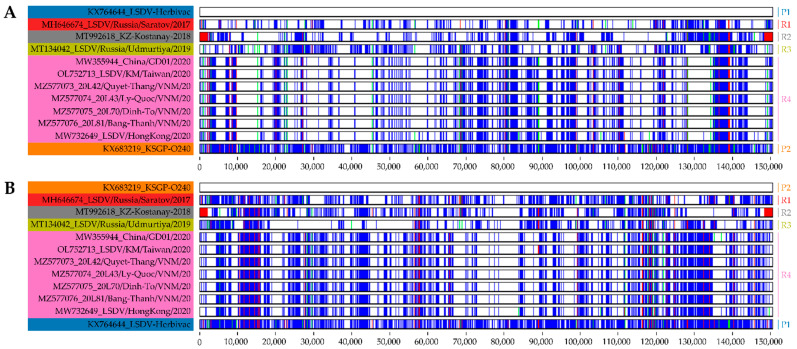
Visual summary of the genomes of the vaccine-like recombinant strains and their putative parental strains. Each panel shows a base-by-base comparison of the different sequences with the top sequence that served as the reference. The recombinant strains were compared to both a Neethling-based vaccine strain (**A**) and a KSGP-based vaccine strain (**B**). The following colouring scheme was used: perfect match (white), SNP (blue), deletion (red) and insertion (green). P1: parent 1, P2: parent 2, R1: recombinant group 1, R2: recombinant group 2, R3: recombinant group 3, R4: recombinant group 4.

In the last five years, several vaccine-like recombinant LSDV strains have emerged throughout Asia. To visualise the relationships between the putative parental strains and the recently discovered recombinant strains, we compared their genomes using Base-By-Base. As shown in Figure 2, the genomes of the recombinant strains appeared to consist of a patchwork of DNA fragments derived from Neethling- and KSGP-like LSDV strains. Based on the distribution of SNPs and InDels, the recombinant strains can be divided into at least four different groups: R1 to R4 (Figure 2 and Figure S1). A pairwise comparison between the putative parental strains and the recombinant strains revealed only a limited number of unique positions that were not found in either of the parental strains. The lowest number of differences was found in the genomes of the recombinant strains from Kazakhstan, Taiwan and Vietnam, which contained between one and three unique positions. The recombinant strain from Hong Kong differed the most from the parental strains, with 59 unique positions (data not shown).

#### 3.4.2. Reconstruction of the Vaccine-like Recombinant LSDV Strains

Using the same strategy as for the parental LSDV genomes (see Section 3.3.2), we subsequently attempted to reconstruct the recombinant LSDV genomes from B-0517_PCR. Apart from the recombinant strain from Kazakhstan, which was reconstructed after a single round, all recombinant strains required multiple rounds to obtain nearly complete genomes. After the first round, a small number of gaps remained, all of which were located near degenerate or recombinant-specific positions (see Section 3.3.1). All these gaps were filled in the following round(s) thanks to the use of semi-perfect mapped reads.

#### 3.4.3. Recombination Breakpoint Analysis

**Figure 3:**
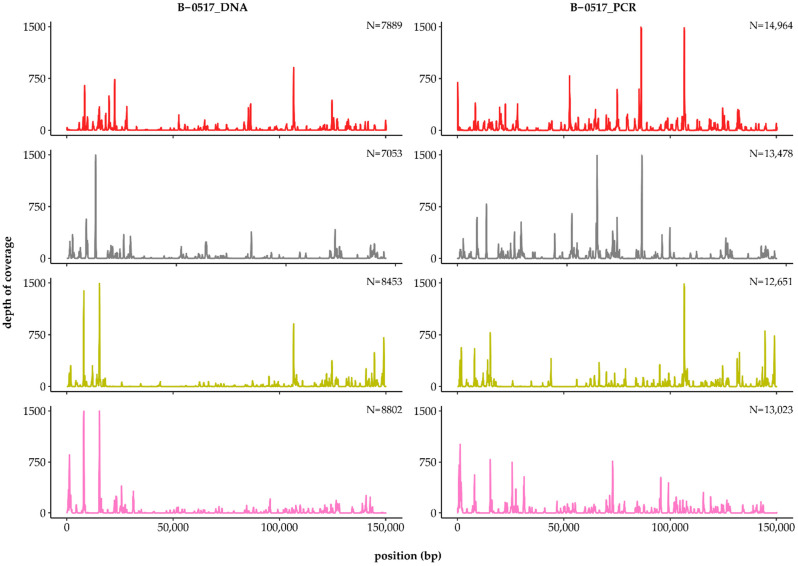
Reads covering potential recombination breakpoints. Reads that mapped perfectly to the GTPV strain or the LSDV vaccine strains were first removed from the B-0517_DNA and B-0517_PCR datasets. Potential recombination breakpoints were subsequently identified by mapping the remaining reads against the genomes of LSDV/Russia/Saratov/2017 (red), KZ-Kostanay-2018 (grey), LSDV/Russia/Udmurtiya/2019 (lime) or 20L42/Quyet-Thang/VNM/20 (pink), allowing no mismatches. The number of paired-end reads is indicated in the top right corner of each subplot.

**Figure 4:**
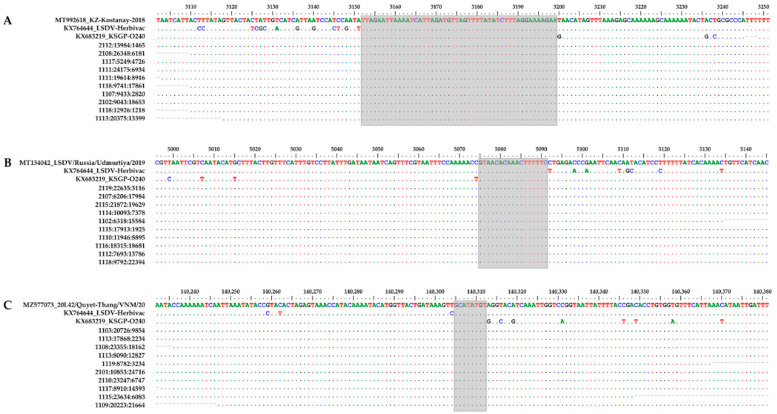
Individual reads covering potential recombination breakpoints. A 150 bp region containing a potential recombination breakpoint in KZ-Kostanay-2018 (**A**), LSDV/Russia/Udmurtiya/2019 (**B**) or 20L42/Quyet-Thang/VNM/20 (**C**) was aligned with both parental strains and 10 reads from the B-0517_DNA dataset. Nucleotides that were identical to the top sequence are shown as dots. Positions refer to the position in the vaccine-like recombinant strain. The putative locations of the recombination breakpoints are highlighted in grey.

The presence of several closely related strains in the same sample complicated the analysis of the LSDV population. Due to the short read lengths of the Illumina sequencing platform, it was expected that most reads would map to both the parental and recombinant strains. To further investigate the presence of the vaccine-like recombinant strains, we decided to focus on the putative breakpoints rather than the whole genomes. To rule out PCR-generated chimaeras, we performed the analysis on both PCR-enriched (B-0517_PCR) and non-enriched (B-0517_DNA) sequencing data. The complexity of the B-0517 datasets was first reduced by removing all reads that perfectly matched the GTPV strain or the LSDV vaccine strains. The remaining reads were then mapped against the genomes of the different vaccine-like recombinant strains, with no mismatches being allowed over the entire read length. Using this approach, we were able to identify a large number of reads scattered across the recombinant genomes in both datasets (Figure 3). Since the recombinant strains contained only a limited number of unique positions, most of the mapped reads either covered an actual breakpoint or lay around a breakpoint, with the R1 read mapping to one parent and the R2 read mapping to the other parent. To illustrate this, we zoomed in on three breakpoint regions from different vaccine-like strains. As can be seen in Figure 4, each breakpoint was covered by at least 10 reads, clearly proving that vaccine-like recombinant strains were already present in B-0517.

## 4. Discussion

From 2017 to 2019, unusual vaccine-like LSDV strains were detected in diseased cattle in Kazakhstan [52] and in neighbouring regions of Russia [26,27] and China [53,54]. Not much later, similar recombinant LSDV strains were found in Taiwan [35], Vietnam [34,55] and Hong Kong [32]. Whole-genome sequencing showed that the two most likely parental strains were a Neethling-like strain (major parent) and a KSGP-like strain (minor parent) [56,57]. Following the emergence of LSDV in Kazakhstan in 2016, the authorities launched a mass vaccination campaign with the Neethling-based Lumpivax vaccine (KEVEVAPI) [36]. As none of the other countries in the affected region used a homologous LSDV vaccine during the same period, it was suggested that the vaccine might have been responsible for the emergence of the recombinant strains [26,33,36]. In a previous study, Haegeman et al. indeed obtained conflicting results when they analysed the Lumpivax vaccine with a series of PCR assays that allowed differentiation between infected and vaccinated animals (DIVA) [40]. Genetic characterisation using Sanger sequencing revealed the presence of Neethling-like, KSGP-like and GTPV-like sequences in the vaccine itself, as well as in samples taken from vaccinated animals. Due to the limited number of genomic regions analysed, the authors could not determine whether the vaccine contained a single recombinant CaPV strain or was a mixture of several CaPV strains. To better determine the exact composition of the Lumpivax vaccine, we performed a genome-wide molecular analysis of two Lumpivax vaccine batches.

To avoid generating additional recombinant strains, we did not perform clonal purification but analysed the vaccine batches directly. Previously, we successfully characterised the genomes of several commercially available Neethling-based vaccine strains as part of a vaccine quality control programme [51]. Although the vaccine strains were from different manufacturers, our analysis revealed only a handful of differences between the genomes. Here, despite sufficient CaPV genome coverage, we were unable to reconstruct a complete genome from either vaccine batch. Instead, the assemblies of B-0517_PCR and B-0219_PCR contained a multitude of shorter contigs that seemed to be derived from different CaPV strains. Indeed, BLAST analysis showed that at least three different CaPVs were present in the Lumpivax vaccine: a Neethling-like LSDV vaccine strain (CaPV subgroup 1.1), a KSGP-like LSDV vaccine strain (CaPV subgroup 1.2) and a Sudan-like GTPV strain (CaPV subgroup 2.3). Although all three viruses were found in both batches, the B-0219_PCR dataset yielded significantly more GTPV contigs than the B-0517_PCR dataset. Based on the number of perfectly mapped reads, B-0219_PCR contained over seven times more GTPV reads than B-0517_PCR. As a result, the number of perfectly mapped LSDV reads dropped from 91% of all reads in B-0517_PCR to 24% of all reads in B-0219_PCR. Despite these large differences, all three CaPV genomes were completely covered in both vaccine batches. These results clearly indicate that the Lumpivax vaccine is not a pure Neethling-based LSDV vaccine but a mixture of several CaPVs. This finding is consistent with the observations of Haegeman et al., who obtained discordant DIVA test results and found both LSDV and GTPV genome fragments [40]. Whether the KSGP-O240 and GTPV strains were added to the Neethling-based vaccine intentionally or accidentally is not known. Nevertheless, the large differences in the composition of both batches clearly show that the production and quality control of the Lumpivax vaccine did not meet the minimum requirements as set out in Chapter 2.3.4 of the Manual of Diagnostic Tests and Vaccines for Terrestrial Animals 2021 (World Organisation for Animal Health) [58]. As described in Chapter 1.1.8, vaccines should be produced in a manner that ensures a uniform and consistent product of high quality [59], which was certainly not the case for these two batches.

Combining multiple closely related viruses increases the risk of recombination, which may occur either naturally in vaccinated animals or in the laboratory during seed production. Although recombination in poxviruses has been well established in vitro, the risk of generating chimeric vaccine/field strains is considered to be low for poxvirus vaccines. However, in contrast to other CaPV vaccines, the Lumpivax vaccine contains multiple virus strains, which increases the probability of co-infection. As far as we know, the recombination frequency and breakpoint patterns have not been studied in animals inoculated with a CaPV mixture. As mentioned above, the vaccine-like recombinant strains could also have been generated in the laboratory during seed production. Recombinant viruses exhibiting a patchy pattern of polymorphic sites have previously been observed in Dryvax, a live attenuated Vaccinia virus (VACV) vaccine that was not clonally purified before seed production [60]. Subsequent in vitro studies showed that recombinant VACV strains exhibited an average of 18 exchanges after a single round of co-infection and that the number of exchanges varied according to the passage history [61]. To gain better insight into the genetic diversity among the recombinant LSDV strains, we aligned the genomes of the putative parental and recombinant strains and visualised the alignment with Base-By-Base. Based on the InDel/SNP signatures, the 10 recombinant strains can be divided into four groups (R1 to R4), each containing a characteristic breakpoint pattern. As shown in Figure 2, each pattern was the result of multiple recombination events, with the number of breakpoints ranging from 126 to 146. The actual number of breakpoints is probably even higher, as the comparison of InDel/SNP patterns does not allow the detection of genetic exchanges in highly conserved regions. The large number of recombinant strains and the large number of breakpoints per genome suggest that the vaccine-like strains arose during seed production.

To confirm that the Lumpivax vaccine was the source of the recombinant strains, we subsequently tried to unravel the complex mixture of viral sequences by gradually reducing the complexity of the datasets. Since the vaccine batches contained not only the parental strains but also multiple recombinant strains, we used an iterative assembly strategy involving in silico enrichment followed by de novo assembly. To reduce the noise generated by the GTPV strain(s), we decided to first reconstruct the more divergent GTPV genome using the B-0219_PCR dataset. Thanks to the high similarity with other GTPV strains of subgroup 2.3, we only needed two rounds of enrichment/assembly to obtain an almost complete GTPV genome. The resulting GTPV-Lumpivax strain was closely related to GTPV-Sudan but contained over 100 SNPs/InDels distributed throughout the entire genome. A comparison with the previously published GTPV genomes of subgroup 2.3 revealed 38 unique signatures that were not present in any of the other available GTPV strains. To reconstruct the different LSDV genomes, we focused on vaccine batch B-0517 as it contained the most LSDV reads. Since chimeric reads can also arise during PCR amplification [40], we created a second dataset directly from purified DNA (i.e., B-0517_DNA). After removing the GTPV reads, we used the same iterative strategy to reconstruct the genomes of the parental and recombinant strains. The genomes of both parental strains were assembled in a single round, indicating that both strains were still present in the vaccine. Although the recombinant strain from Kazakhstan contained a unique signature at position 125,553, we were also able to recover its genome in a single round. Reconstruction of the other recombinant genomes required 2–3 rounds, which means that the reconstructed genomes were not 100% identical to the previously published genomes. Alignment of the published genomes with the scaffolds generated after the first round revealed that all gaps were located near degenerate or recombinant-specific positions. These differences can be explained in several ways. Firstly, some of the recombinant viruses may have emerged from a different vaccine batch than the one analysed in our study. Alternatively, some of the recombinant-specific signatures may have been acquired after the viruses were released in the field. This could be the case for the recombinants discovered in Taiwan and Vietnam, which differed from both parental strains only in 2–3 positions. Finally, the published genomes may contain sequencing errors. It is noteworthy that the published genomes of the recombinant strains from China and Hong Kong contained several degenerate bases, which may indicate that coverage in these regions was insufficient. Regardless of these minor differences, our results clearly show that all breakpoints of the published recombinant strains were present in the B-0517_DNA and B-0517_PCR datasets. Since we used a reference-guided de novo assembly approach, we could only detect breakpoints that had been previously described. The vaccine could thus contain other recombinant strains that have not yet been detected in the field. Recently, Saltykov et al. used a typing method based on the G-protein-coupled chemokine receptor to reveal an unprecedented level of biodiversity among Russian LSDV strains isolated between 2017 and 2019 [62]. Unfortunately, none of these strains has been characterised at the genome level. Additional experiments using a long-read sequencing platform (e.g., PacBio’s HiFi reads) are required to fully explore the diversity present in the Lumpivax vaccine. Nevertheless, the fact that numerous breakpoints were detected in the vaccine and that virus-like strains containing the same breakpoints emerged in the field shortly after vaccination provides sufficient evidence to conclude that the recombinant strains were already present in the vaccine.

## 5. Conclusions

In this study, we performed a genome-wide molecular analysis of two Lumpivax vaccine batches. Contrary to the label, the Lumpivax vaccine is not a pure Neethling-based LSDV vaccine but a complex mixture of several CaPVs. Using an iterative enrichment/assembly strategy, we obtained the complete genomes of a Neethling-like LSDV vaccine strain (CaPV subgroup 1.1), a KSGP-like LSDV vaccine strain (CaPV subgroup 1.2) and a Sudan-like GTPV strain (CaPV subgroup 2.3). The same analysis also revealed the presence of several recombinant LSDV strains that were (nearly) identical to the recently described vaccine-like LSDV strains. Based on the InDel/SNP signatures, the vaccine-like recombinant strains can be divided into four groups, each with a distinct breakpoint pattern. The large number of recombinant strains and the large number of breakpoints per genome suggest that the vaccine-like strains arose during seed production and not in the field. Although further research is needed, the emergence of vaccine-like LSDV strains in large parts of Asia thus appears to be the result of a spillover from vaccinated animals. Our results show once again the importance of an adequate and independent quality control programme covering the entire vaccine manufacturing process. Finally, our study demonstrates the power of WGS/HTS-based typing. Despite using a short-read sequencing platform, we were able to unravel the complex mixture of closely related viral strains contained in the Lumpivax vaccine. Newly developed long-read technologies will make this type of analysis even more robust. Even though several challenges still need to be addressed, HTS is a powerful tool that should be integrated more often into future quality control programmes.

## Figures and Tables

**Table 1:** Summary of the main output metrics for datasets B-0517_DNA, B-0517_PCR and B-0219_PCR.

Output Metric	B-0517_DNA	B-0517_PCR	B-0219_PCR
Paired-end raw reads	3,526,949	3,176,585	3,106,361
Paired-end reads after quality trimming	3,512,424	3,149,127	3,073,447
Proportion of reads mapping to NI-2490 ^1^ (%)	83.29	99.66	99.50
Proportion of NI-2490 ^1^ genome covered (%)	100.00	99.89	99.86

^1^ Lumpy skin disease virus NI-2490 (NC_003027).

**Table 2:** Quality metrics of the de novo assemblies of the B-0517_PCR and B-0219_PCR datasets. Both datasets were assembled using a standard de novo strategy, and the assemblies were evaluated with QUAST using a typical Neethling-based vaccine strain (i.e., LSDV-Herbivac) as a reference genome.

Quality Metric	B-0517_PCR	B-0219_PCR
Genome fraction (%)	90.169	94.091
Duplication ratio	1.058	1.092
GC (%)	25.82	25.24
Contigs	126	45
Contigs (≥1000 bp)	51	31
Contigs (≥5000 bp)	1	14
Contigs (≥10,000 bp)	0	5
Mismatches per 100 kbp	279.32	2202.11
InDels per 100 kbp	21.59	124.14
LGA50 ^1^	35	7
NGA50 ^2^	1370	6783

^1^ Minimal number of aligned fragments that cover half of the reference genome. ^2^ Shortest length among the LGA50-aligned fragments.

## Data Availability

The raw data from B-0517_DNA, B-0517_PCR and B-0219_PCR have been submitted to the SRA under BioProject number PRJNA835231.

## Supplementary Materials

The following supporting information can be downloaded at: https://www.mdpi.com/article/10.3390/v14071429/s1, Table S1: BLAST analysis of contigs obtained with a standard de novo assembly without in silico enrichment; File S1: Putative genome sequence of the GTPV strain found in the Lumpivax vaccine; Figure S1: Visual summary of the genomes of the vaccine-like recombinant strains found in the Lumpivax vaccine and their putative parental strains.
