# Supplementary figures and images for: Recombinant LSDV Strains in Asia: Vaccine Spillover or Natural Emergence?
Source: Viruses. 2022 Jun 29;14(7):1429. doi: 10.3390/v14071429 (PMC9318037; doi:10.3390/v14071429)

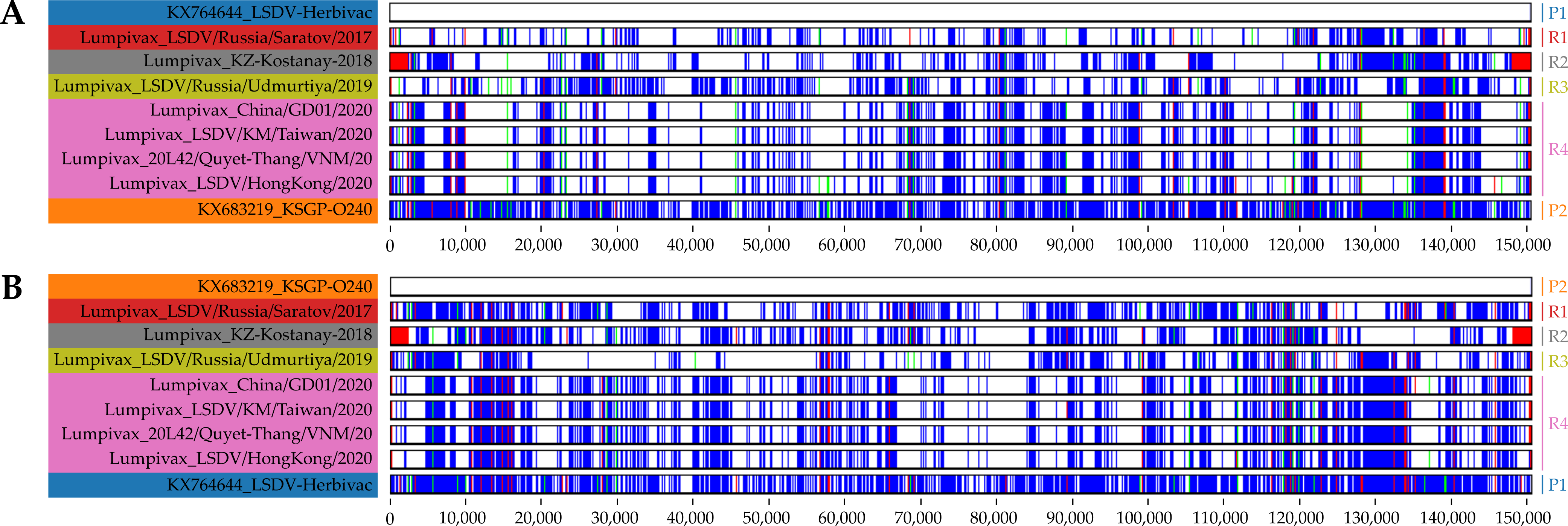

Supplement: Supplementary file 1 [file viruses-14-01429-s001.zip › Figure S1.png]
